# Your next clinical cancer research project: preparation in a multidisciplinary environment is key

**DOI:** 10.3332/ecancer.2017.ed64

**Published:** 2017-03-14

**Authors:** Mieke Van Hemelrijck, Cecilia Bosco

**Affiliations:** King’s College London, Division of Cancer Studies, Cancer Epidemiology Group, London, SE1 9RT, UK

**Keywords:** multidisciplinary research, translational research, good research practice

## Abstract

For clinical cancer research to have an impact, careful planning in a multidisciplinary translational setting is required. Once your multidisciplinary research team is established, the first step is to define an answerable research question, followed by planning the study design and identifying the study population—allowing for appropriate statistical analyses of high-quality data, whether patient or lab-based. Finally, interpretation of the results also requires multidisciplinary discussions and academic writing in a clear and concise way. This editorial is designed to give you some helpful tips and structure when planning your next clinical cancer research project.

Are you keen to make a difference to cancer patients? Do you have brilliant ideas for new translational research projects? Are you thinking about getting a better understanding of the patient pathway in your hospital? Or do you want to assess patient outcomes following a new treatment procedure?

Then you are ready to start a clinical research project! However, for research to have an impact, careful study planning is required to ensure that all data, whether patient or lab-based, can be analysed according to recognised standards and that findings can be published in peer-reviewed scientific journals.

This editorial is a guideline based on our experience as clinical and molecular cancer epidemiologists who are based in a multidisciplinary research environment where both lab-based scientists and clinicians ask for (statistical) help with their research projects. While each research project is different and may require different approaches, planning and multidisciplinary collaboration are undoubtedly the two most important components of a successful clinical cancer research project. The following highlights a few steps that we have found useful when conducting clinical cancer research.

## Define the research question whilst engaging with a multidisciplinary research team

In cancer care the multidisciplinary team has been developed to ensure that patients receive timely treatment and care from appropriately skilled professionals with adequate support. The team is there to monitor adherence to clinical guidelines and promote effective use of resources [1]. However, in the field of clinical cancer research a multidisciplinary approach refers to a coherent translational team—including those who are part of the multidisciplinary care team as well as scientists, epidemiologists, bioinformaticians, biostatisticians, and potentially also health economists, patient representatives, and policy makers.

The first thing the team needs to do is define the research question or the main aim of the project. This may sound trivial, but it has to be ensured that the question has not yet been answered in a definite way. You may want to consider answering a specific question in a different study population (e.g. different age group) or to validate the methods used by other researchers. Several frameworks exist to help you phrase an answerable research question; PICOT is well-known in the field of evidence-based medicine and suggests that a clinical research question can be divided into five components: Population/patient, Intervention/indicator, Comparator/control, Outcome, and the Timeframe [2].

The research work that will answer your question has to be conducted, so that it can contribute to current medical practice or be compared with existing work. This will then provide further insight into the field you are researching; may influence patient care or health services; and can help attract future funding. It is therefore essential to conduct a literature review and identify the current status of the research area.

Finally, when planning your research project/question it is important to consider and apply for ethical approval. No study can be conducted or published without relevant ethical permissions [3].

## Outline the study population and data required to answer the question

A definition of the study population in which the research question can be studied is required. A study population does not necessarily refer to patients. Most clinical epidemiological studies will be population-based, however also lab-based scientists may have to think about how they will populate their data set—whether they are looking at cell-lines, tumour or blood samples, mice, etc. If the research is focused on a population-based study, it is important that the cohort is clearly defined and representative of a general population relevant to the research question. Selection mechanisms that may harm the internal validity of the study need to be avoided. For instance, if you want to study the effects of red meat intake on risk of pancreatic cancer, there may be issues with selection mechanisms if those who fill out your questionnaire are very different from those who refuse to participate in your study. Furthermore, the dataset needs to include all the information required to answer the predefined research question.

To define the right study population, whether population or lab-based, it is helpful to think about the following questions [4, 5]:

1. What is the exposure of interest (e.g. patient characteristic, expression levels of a biomarker)?
2. What is the outcome of interest (e.g. cancer-specific death, evidence of cell death)?
3. What information is required about variables that may affect the association you are studying (e.g. sex, different concentrations of a medium)?
4. Given the nature of the study, would it be feasible to do a cohort study or case-control study? Or is there another study design that is more appropriate (e.g. small pilot study)?
5. Will your study design allow for studying something new or for comparing your results with existing findings?

The quality of your data is thus important. Depending on the type of study, it may for instance also be of interest to look into development and use of validated questionnaires [6].

## Data collection

Next, procedures have to be put in place to collect the required data. Standard operating procedures (SOPs) are usually required as part of the ethical approval process, whether this refers to tissue preparation, collection of blood samples, or collection/entering of clinical patient data—which may or may not involve a patient consent and a variety of team members (e.g. research nurses, biobank technicians, clinical trial coordinators). These SOPs will help when writing the Methods section of the related scientific paper.

It is also helpful to create a dataset that makes it straightforward to conduct statistical analyses. As epidemiologists, we find it helpful when we provide our colleagues in the clinic or lab with the following guidelines:

1. One row per observation whether it is a patient, tissue sample, cell line, etc
2. One column per variable of interest
3. Avoid text values for the variables and ensure that all categorical variables are coded consistently using numbers. For instance: 0 = no chemotherapy; 1 = chemotherapy with taxanes; 2 = chemotherapy without taxanes.
4. Ensure that all dates are entered as dates in a consistent way
5. Give clear, short and self-explanatory names to each column
6. Provide the person analysing your data with an anonymised file

In this era of big data, more reports are being developed to guide researchers and help better define how to extract value from data, create analytical tools to enhance utility of data, and develop data science concepts and tools for all stakeholders involved in translational research [7].

## How will you analyse the data collected?

This will involve planning of experimental settings and/or the epidemiological/statistical methods. It is recommended you think about the following questions [4]:

1. Given the study design, what would be the preferred measure of association?
2. How will the exposure and outcome of interest be measured?
3. How will variables affecting the link between exposure and outcome be taken into account?
4. Is there a need to do sensitivity analyses or experiments in different settings?

If you require statistical assistance throughout your project, it is helpful to prepare template figures and tables beforehand as that will make the communication between different disciplines (e.g. biostatisticians, clinicians, and lab-based scientists) more straightforward.

## Write a manuscript

Once all the results are in place, it is important to identify those that are clinically or scientifically relevant so that a coherent story can be put together. This is another key time in your project where multidisciplinary collaboration and discussion is required. It should help you identify the main message that you want to convene in your manuscript.

Many useful publications exist to guide you with writing scientific publications [8]. It is best to communicate as clearly as possible, in a style appropriate for serious academic work, but avoiding the use of difficult sentence constructions wherever possible. Simple, short sentences are generally best. There is much to be gained from critiquing your own work. The following structure can help with writing scientific manuscripts:

1. Introduction
  This section provides a background to the work. The introduction usually builds up to a final paragraph in which the main aim of your study is explained.
2. Methods
  Usually, this section first provides a description of the study population and the data collection, followed by a detailed overview of the experimental methods or statistical analyses conducted.
3. Results
  These should be written in a similar order to the methods described in the Methods section. Ideally, each paragraph describes a different table or figure—they have to come in a logical order, which needs to be described clearly to the reader. There is no need to repeat everything that is shown in the tables or figures, but it is important to highlight the important points (and otherwise refer to the tables and figures). The tables and figures also need self-explanatory captions, as a reader should be able to understand them without reading the actual manuscript. Make sure that tables and figures illustrate different study findings.
4. Discussion
  There are several ways to structure a Discussion section, but the following suggestion has turned out to be helpful in many of our rather complex papers [9]. The first paragraph is a summary of the findings. The next one or two paragraphs then describe what is currently provided in the literature—giving slightly more detailed information than what has been provided in the Introduction. This is then followed by some paragraphs interpreting the study findings in relation to what is already known. Before the concluding paragraph, authors often describe the strengths and limitations of their study.
5. Conclusion
  The conclusion should not be a repetition of the first paragraph of the Discussion, but should focus on the main finding and its potential clinical impact with recommendations for future studies or practice.
6. References
  Using bibliographic software will allow for formatting of the references according to the preferred style of the journal. Make sure to use established scientific resources for your references.

Moreover, several guidelines have been developed to help structure a manuscript (and as a result study design), of which the STROBE checklist [10] and CONSORT guidelines [11] are good examples. The first one is designed to strengthen the reporting of observations studies in epidemiology, whether the latter focuses on consolidated standards of reporting trials. More guidance on designing and reporting results from translational research can be found in Rubio *et al* [12].

The above is a guideline based on our own experience—though we are aware that each clinical cancer research project has its own nuances. Nevertheless, in the many different research projects with which we have been involved, careful planning in an open and honest multidisciplinary research environment has always proven to be very rewarding, both in terms of research findings as well as development of personal research skills.
